# Exploring the impact of event-related and personal vulnerability characteristics on mental health after pandemic-related trauma exposure

**DOI:** 10.1080/20008066.2026.2700829

**Published:** 2026-07-31

**Authors:** Anouk Van Duinkerken, Mark W.G. Bosmans, Imme J. Rahmon, Elske Marra, Christos Baliatsas, Michel L.A. Dückers

**Affiliations:** a Faculty of Behavioural and Social Sciences, University of Groningen, Groningen, the Netherlands; b Disasters and Environmental Hazards, Nivel (Netherlands Institute for Health Services Research), Utrecht, the Netherlands; c Department of Health, Living Environment, and Aftercare, National Institute for Public Health and the Environment, Bilthoven, the Netherlands; d ARQ Center of Expertise on the Impact of Disasters and Crises, ARQ National Psychotrauma Centre, Diemen, the Netherlands

**Keywords:** Posttraumatic stress symptoms, pTSD, trauma, mental wellbeing, mental health, COVID-19, Síntomas de estrés postraumático, COVID-19, bienestar, TEPT

## Abstract

**Background:** The COVID-19 pandemic heightened exposure to potentially traumatic events (PTEs), such as severe illness or the loss of a loved one. Its prolonged nature may have influenced how mental health symptoms evolved.

**Objective:** This study examined changes in posttraumatic stress symptoms (PTSS) and mental wellbeing over time and identified factors contributing to differences in these outcomes.

**Method:** Panel survey data were collected from Dutch youth and adults between March 2022 and June 2024. Participants reported PTEs during the pandemic and whether they still felt affected by these events. PTSS were measured with the PCL-5, anchored to their most distressing event. Mental wellbeing was assessed using the MHI-5. Mixed-effects models examined how risk factors influence severity of PTSS and mental wellbeing. Qualitative analysis of an open-ended question provided insight into the influence of the COVID-19 pandemic on how participants’ coped with PTEs.

**Results:** 5,782 observations (of 3,445 individuals) were analysed. PTSS declined slightly over time, but personal vulnerability factors were more strongly associated with PTSS than event-related factors. For mental wellbeing, only personal vulnerability factors were significant covariates. Thematic analysis of the open question revealed four main themes: lack of social contact and support, (interference with) mourning rituals, fear and uncertainty surrounding infection, and renewed appreciation for life and relationships with others.

**Conclusions:** Our findings indicate that personal vulnerability factors and the broader pandemic context, which disrupted normal social coping mechanisms, played a prominent role in the aftermath of pandemic-related trauma. In future public health crises, policies should aim to maintain social connectedness and emotional support to promote psychological recovery.

## HIGHLIGHTS

- Posttraumatic stress symptoms declined slightly over time in Dutch youth and adults exposed to pandemic-related traumatic events.
- Personal vulnerability factors showed stronger associations with posttraumatic stress symptoms and mental wellbeing than event-related factors.
- The pandemic disrupted normal coping processes by hindering social support, mourning rituals, and increasing fear and uncertainty.

## Abstract (es)

**Antecedentes:** La pandemia de COVID-19 aumentó la exposición a eventos potencialmente traumáticos (PTEs en su sigla en inglés), como una enfermedad grave o la pérdida de un ser querido. Su naturaleza prolongada pudo haber influido en cómo evolucionaron los síntomas de salud mental.

**Objetivo:** Este estudio examinó los cambios en los síntomas de estrés postraumático (SEPT) y el bienestar mental a lo largo del tiempo, identificando factores que contribuyen a las diferencias en estos resultados.

**Método:** Se recopilaron datos de encuesta panel de jóvenes y adultos holandeses entre marzo de 2022 y junio de 2024. Los participantes informaron los PTEs durante la pandemia y si aún se sentían afectados por estos eventos. Los SEPT se midieron con el PCL-5, anclados a su evento más angustiante. El bienestar mental se evaluó utilizando el MHI-5. Los modelos de efectos mixtos examinaron cómo los factores de riesgo influyen en la gravedad de los SEPT y el bienestar mental. El análisis cualitativo de una pregunta abierta proporcionó una visión sobre la influencia de la pandemia de COVID-19 en cómo los participantes afrontaron los PTEs.

**Resultados:** Se analizaron 5.782 observaciones (de 3.445 individuos). Los SEPT disminuyeron ligeramente con el tiempo, pero los factores de vulnerabilidad personal se asociaron más fuertemente con los SEPT. Para el bienestar mental, solo los factores de vulnerabilidad personal fueron covariables significativas. El análisis temático de la pregunta abierta reveló cuatro temas principales: falta de contacto social y apoyo, (interferencia en) los rituales de duelo, miedo e incertidumbre en torno a la infección, y renovada apreciación por la vida y las relaciones con los demás.

**Conclusiones:** Nuestros hallazgos indican que los factores de vulnerabilidad personal y el contexto pandémico más amplio, que alteraron los mecanismos sociales normales de afrontamiento, desempeñaron un papel destacado tras el trauma relacionado con la pandemia. En futuras crisis de salud pública, las políticas deberían buscar mantener la conexión social y el apoyo emocional para promover la recuperación psicológica.

## 1. Introduction

In March 2020, the World Health Organization (WHO) declared COVID-19 a global pandemic (WHO, 2020). Since then, the pandemic has resulted in nearly 800 million confirmed cases and over 7 million deaths worldwide (WHO, n.d.). In response, governments implemented various public health measures, including lockdowns, quarantine regulations, and social distancing policies (Hale et al., 2021). These measures, along with the broader consequences of the pandemic, increased prolonged exposure to potentially traumatic events (PTEs), such as severe illness, the death of loved ones, and work-related stress, particularly among healthcare workers.

Public health measures such as lockdowns, social distancing, and the closure of public spaces limited access to many commonly used coping resources after experiencing a PTE, including social support networks, community activities, and religious or cultural practices (Holmes et al., 2020; Tunçgenç et al., 2023). Studies have shown that reduced access to social interaction and professional help during the pandemic was associated with higher levels of psychological distress and delayed recovery from traumatic events (Ogueji et al., 2021; Vindegaard & Benros, 2020). Additionally, the chronic stress and prolonged uncertainty surrounding the pandemic may have depleted individuals’ coping capacities over time, making them more vulnerable to the mental health consequences of potentially traumatic events (Manchia et al., 2022; Pfefferbaum & North, 2020).

Trauma-related mental health encompasses a range of psychological difficulties that can arise following exposure to potentially traumatic events. The most commonly studied trauma-related mental health outcome is post-traumatic stress disorder (PTSD), but trauma exposure can also lead to anxiety disorders, depressive disorders, substance use problems or complicated grief (Drucker, Levi-Belz & Hamdan, 2025; Norris et al., 2002; Overstreet et al., 2017). These conditions often co-occur and can have serious consequences, including impaired daily functioning and reduced quality of life (Feriante & Sharma, 2023).

While cross-sectional studies have consistently reported elevated rates of trauma-related mental health problems during the early stages of the COVID-19 pandemic (van Duinkerken et al., 2025), research into longer-term outcomes remains limited. The pandemic was not a singular or acute event, it was a prolonged crisis marked by ongoing uncertainty, repeated stressors, and disrupted access to key resources such as social contact and health care (Horesh & Brown, 2020; Pfefferbaum & North, 2020). The impact unfolded gradually, with effects that may surface later, persist or fluctuate over time. This highlights the importance of longitudinal research to capture the evolving nature of trauma-related symptoms and wellbeing. Although initial findings suggest that symptoms may subside over time (van Duinkerken et al., 2025), there is a lack of insight into how the symptoms evolve over a longer timeframe and how both characteristics of the event and individual vulnerabilities contribute to persistent psychological distress or reduced wellbeing.

The psychological impact of trauma varies considerably across individuals, depending on both characteristics of the traumatic event and personal vulnerability factors (Bonanno et al., 2010; Tortella-Feliu et al., 2019), which fits the ecological model theory (Bronfenbrenner, 1979; Harvey, 1996). Event-related risk factors include the type, intensity, and duration of exposure. On the personal vulnerability, factors such as pre-existing mental health problems, limited social support, concurrent life stressors and female gender have consistently been associated with poorer post-trauma outcomes (Tortella-Feliu et al., 2019). Understanding how these factors interact is essential to identify who is most at risk for lasting psychological effects.

Traumatic and stressful life events rarely occur in isolation and are often embedded within broader social and environmental contexts. Large-scale crises, such as the COVID-19 pandemic, provide a unique opportunity to examine how event characteristics and individual vulnerability factors jointly shape mental health outcomes. Importantly, although the COVID-19 pandemic represents a specific global crisis, it shares key features with other large-scale disruptive events, such as natural disasters, public health emergencies, and periods of prolonged societal disruption. Studying mental health responses in this context therefore contributes to a broader understanding of psychological adaptation under conditions of widespread stress. In particular, identifying whether personal vulnerability factors or event-related characteristics play a stronger role has implications for prevention and intervention strategies across different crisis situations.

The present study uses quantitative and qualitative data from a panel survey of the general population in the Netherlands. Specifically, we aim to assess changes in posttraumatic stress symptoms and mental wellbeing over time following exposure to COVID-19-related traumatic events. Additionally, we are interested in the relative contributions of event-related characteristics and personal vulnerability factors in explaining individual differences in long-term mental health outcomes. In addition, we explore participants’ open-ended reflections to gain insight into how the unique circumstances of the pandemic may have shaped their coping process.

## 2. Methods

### 2.1. Participants

Survey data were collected via online questionnaires through the panel bureau IPSOS I&O between March 2022 and June 2024. The panelists are recruited through random sampling from population registers or large address databases. In this study, two groups were approached: youth (12–25 years old) and adults (26+). Youth aged 12–16 were invited through adult panel members with children living at home, while those aged 17 and older were contacted directly.

### 2.2. Procedures and measures

Exposure to COVID-19 related PTEs was measured with a pre-defined list of experiences/events that could have occurred during the pandemic. This list was compiled by disaster health researchers and public health experts from the regional public health services, and included the following events:

- I was hospitalised due to COVID-19.
- Someone important to me was hospitalised due to COVID-19.
- Someone important to me passed away from COVID-19.
- I was afraid that I or someone important to me would get COVID-19.
- In my work, I saw many people who were seriously ill or passed away from COVID-19.
- Due to COVID-19 measures, I was unable to provide support or care to someone important to me.
- Due to COVID-19 measures, I was unable to say goodbye to someone who passed away.
- I experienced threats and/or violence due to discussions about COVID-19 measures.

For each of these events, respondents were also asked to answer whether they still felt affected by that event. In case the respondents still felt affected by multiple events, they had to choose their most distressing one, which would be used as the index event to which the PTSS measure would be anchored to. They also answered how long it has been since they experienced that event, ranging from 1 (*less than a month*) to 4 (*more than a year*).

PTSS were assessed using the PTSD Checklist for DSM-5 (PCL-5), a 20-item self-report measure that evaluates the severity of PTSS based on DSM-5 criteria (Weathers et al., 2013). Participants rated the extent to which they were bothered by each symptom in the past month on a 5-point Likert scale (0 = *Not at all* to 4 = *Extremely*), with higher scores indicating greater PTSS severity. Probable PTSD diagnosis on the PCL-5 can be determined by applying the DSM-5 cluster criteria, requiring at least one intrusion and avoidance symptom, two negative alterations in cognition and mood, and two arousal symptoms rated ≥2 (‘moderately’ or higher).

Mental wellbeing was assessed using the Mental Health Inventory-5 (MHI-5), a brief measure of psychological distress and wellbeing (Berwick et al., 1991). The MHI-5 consists of five items rated on a 6-point scale (1 = *All of the time* to 6 = *None of the time*), with total scores ranging from 0 to 100, where higher scores indicate better mental health.

Loneliness was measured using the De Jong-Gierveld Loneliness Scale, a validated instrument designed to assess emotional and social loneliness (De Jong-Gierveld & van Tilburg, 2006). The scale consists of 6 items, with responses ranging from 0 (*not lonely*) to 6 (*severely lonely*), where higher scores indicate greater perceived loneliness.

Stress was assessed using seven items referring to different sources of stress experienced over the past four weeks (e.g. work/study, home situation, social contacts). Participants indicated how often they felt stressed about each source on a five-point scale ranging from *never* (1) to *very often* (5). A stress sum score was computed by counting the number of sources for which participants reported feeling stressed *often* or *very often*.

Physical symptoms were measured with eleven items assessing physical and psychological complaints over the past four weeks (e.g. headaches, sleep problems, fatigue), rated on the same five-point scale ranging from never to very often. This list is based on the validated Symptoms and Perceptions (SaP) checklist (Yzermans et al., 2016). A somatic complaints sum score was calculated by counting the number of symptoms reported *often* or *very often*.

Educational level was categorised according to the ISCED classification. Low education included ISCED 0–2 (no education, primary education, or lower secondary vocational education). Medium education included ISCED 3–4 (general secondary education, such as havo or vwo, and upper secondary vocational education, mbo levels 2–4). High education included ISCED 5–6 (higher professional education, hbo, and university-level education, wo, including bachelor's and master's degrees).

To explore how the COVID-19 pandemic affected the processing of participants’ most distressing event, an open-ended question was included in the fifth round of the panel survey (September 2022): *How has the COVID-19 period influenced your ability to cope with this event?* This supplementary, exploratory analysis was conducted to provide further context and nuance to the quantitative findings. The aim was to complement and help interpret the quantitative findings by incorporating lived experiences about the influence of the pandemic on coping with events.

### 2.3. Data analysis

#### 2.3.1. Quantitative analysis

Data were analysed using mixed-effects linear regression models to account for the hierarchical structure of repeated measures nested within individuals. Separate models were run for different outcome variables: PTSS and mental wellbeing, both measured as a continuous sum score. All models included a random intercept for participant ID to adjust for within-person clustering. Robust standard errors were applied to account for potential heteroskedasticity and violations of normality assumptions.

Two models were estimated. The initial model (Model 1 for PTSS and Model 3 for mental wellbeing) included event-related factors: number of measurement timepoints since exposure, time since exposure to the most distressing PTE, time between measurement timepoints, and the type of potentially traumatic event. The event with the lowest mean score on the outcome is used as the reference category. This approach enables all comparisons to be made relative to the event associated with the least psychological impact in the sample. By doing so, the estimated coefficients for other event types represent the expected difference in outcome scores compared to this low-impact baseline, making it easier to interpret which events are associated with higher levels of distress. The extended model (Model 2 for PTSS and Model 4 for mental wellbeing) additionally included personal vulnerability factors: loneliness, perceived stress, physical symptom burden, age, gender, and educational level. Multicollinearity was assessed for the full model by calculating variance inflation factors, which indicated no evidence of problematic multicollinearity. In addition, we examined the bivariate associations between the covariates, which did not suggest concerning overlap.

Nonlinearity in the associations between age, measurement time points, and the outcomes was tested by adding quadratic and cubic terms. If these higher-order terms were significant (*p* < .05) based on joint significance tests they were retained in the model; otherwise, only the linear term was included.

Model fit was compared using Akaike’s Information Criterion (AIC), Bayesian Information Criterion (BIC), and log-likelihood values (LL). Lower AIC and BIC values and a higher LL were used as indicators of better model fit. Due to the use of robust standard errors, likelihood ratio tests were not conducted. As a sensitivity analysis, we estimated multilevel cross-lagged models to examine the temporal directionality between time-varying vulnerability factors and mental health outcomes. These models tested lagged associations in both directions across adjacent waves while accounting for repeated observations within individuals.

#### 2.3.2. Qualitative analysis

Responses to the open-ended question were analysed using thematic analysis. This exploratory analysis was conducted as a complement to the quantitative data. An inductive approach was applied: responses were read repeatedly to gain familiarity with the material, meaningful segments were coded, and related codes were grouped into preliminary themes. These themes were subsequently reviewed and refined, and representative quotes were selected to illustrate the findings. The results contribute to contextualising and deepening the interpretation of the observed statistical associations.

## 3. Results

### 3.1. Descriptive information

Out of a total dataset comprising 62,003 observations, the PCL-5 was completed in 5,782 observations. Therefore, these were included in the analyses. In total, 3,445 unique individuals completed the PCL-5 at least once. The majority of these respondents completed the measure on a single occasion (*N* = 2,021), while 883 completed it twice, and 541 completed it three or more times. 61.7% identified as female and 38.3% as male. Most participants were aged between 18 and 75 years, with the largest age groups being 56–65 years (20.8%) and 66–75 years (19.4%). Regarding educational level, 34.8% had a low level of education, 33.1% a middle level, 13.1% a high level, and 18.9% were still in education. Based on the short De Jong Gierveld scale, 42.8% of respondents were not lonely, 33.8% were moderately lonely, and 23.3% were severely lonely. The mean scores for experiencing somatic symptoms and stress often to very often were 2.24 (SD = 2.35) and 1.08 (SD = 1.49) respectively (Table 1).

**Table 1.** Demographic characteristics and measures of (psychosocial) wellbeing indicators (*N* = 5,782).

Variable	Level / Measure	% or M(SD)
Gender	Men	38.3
	Women	61.7
Mean age in years		49.4 (19.6)
Education level^a^	Currently in education	18.9
	Low	34.8
	Middle	33.1
	High	13.1
Loneliness^b^	Not lonely	42.8
	Moderately lonely	33.8
	Severely lonely	23.3
PTSS^c^	PCL-5 (0–80)	15.6 (13.7)
Mental wellbeing^d^	MHI-5 (0–100)	66.1 (17.8)
Somatic symptoms	Count of complaints (very) often experienced in the past 4 weeks (0–11)	2.2 (2.4)
Stress	Count of factors causing the respondent to feel stressed (very) often (0–7)	1.1 (1.5)

Note:

^a^ Education levels are based on ISCED 2011. 0–2 = Low, 3–4 = Middle, 5–6 = High, ‘Currently in education’ = enrolled in any level of education (irrespective of ISCED level).

^b^ Loneliness measured with the short De Jong-Gierveld scale

^c^ PTSS symptoms in the last 4 weeks measured with PCL-5 = PTSD Checklist for DSM-5

^d^ Mental wellbeing in the last month measured with the MHI-5 = Mental Health Inventory.

### 3.2. PTSS

The average PCL-5 score was 15.58 (SD = 13.74). 13.49% had probable PTSD based on the DSM-5 scoring. See the appendix for average prevalence of probable PTSD per round.

#### 3.2.1. Event related covariates

Multilevel linear regression models were used to assess the impact of event-related characteristics and personal vulnerability characteristics on PTSS severity. In Model 1 (Table 2), later measurement timepoints (coefficient = −0.93, 95% CI: −1.30 to −0.56) and longer time since the exposure to the most distressing event (coefficient = −0.66, 95% CI: −1.01 to −0.30) were both significantly associated with lower PTSS severity. Participants who had been hospitalised due to COVID-19 reported higher symptom severity compared to those who had someone important to them hospitalised (coefficient = 5.15, 95% CI: 0.97–9.34). Being unable to provide support or care to someone due to COVID-19 measures (coefficient = 4.25, 95% CI: 2.35–6.14), and experiencing threats and/or violence related to discussions about COVID-19 measures (coefficient = 6.90, 95% CI: 4.64–9.17), were both associated with elevated PTSS. Other event types were not significantly associated with symptom severity. The between-person variance was estimated at 118.19 (95% CI = 108.12–129.21), while the within-person variance was 56.94 (95% CI = 51.80–62.59).

**Table 2.** Mixed effects linear regression: Event-related covariates in relation to PTSS (Model 1).

Variable	Coef.	Std. Error	*p*-value	95% CI	
Fixed effects					
Timepoint	−0.93	0.19	.00^a^	−1.30	−0.56
Days between timepoints^a^	0.00^a^	0.00^a^	.47	−0.00^a^	0.00^a^
Time since exposure to the most distressing PTE	−0.66	0.18	.00^a^	−1.01	−0.30
Exposure to PTE					
Someone important to me was hospitalised due to COVID-19	Ref.^b^				
Own hospitalisation due to COVID-19	5.15	2.13	.02	0.97	9.34
Someone important to me passed away from COVID-19	1.11	0.93	.23	−0.71	2.92
I was afraid that I or someone important to me would get COVID-19	1.09	0.85	.20	−0.59	2.76
In my work, I saw many people who were seriously ill or passed away from COVID-19	0.64	1.24	.61	−1.79	3.06
Due to COVID-19 measures, I was unable to provide support or care to someone important to me	4.25	0.97	.00^a^	2.35	6.14
Due to COVID-19 measures, I was unable to say goodbye to someone who passed away	1.57	0.89	.08	−0.18	3.32
I experienced threats and/or violence due to discussions about COVID-19 measures	6.90	1.16	.00^a^	4.64	9.17
Constant	15.92	0.99	.00^a^	13.98	17.86
Random effects (variance)					
Between persons	118.19	5.37		108.12	129.21
Within-person	56.94	2.75		51.80	62.59

Note:

^a^ Value < 0.00 (not shown due to rounding).

^b^ Reference category.

#### 3.2.2. Personal vulnerability factors

In Model 2 (Table 3), which included both event-related and personal vulnerability covariates, several associations with PTSS severity were observed. Consistent with Model 1, experiencing threats or violence related to COVID-19 measures remained a strong association with PTSS (coefficient = 4.74, 95% CI: 2.74–6.73), as did being unable to provide care or support due to COVID-19 measures (coefficient = 2.17, 95% CI: 0.57–3.77). In later measurements, symptom levels were slightly lower (coefficient = –0.61, 95% CI: –0.95 to –0.27), and time since exposure remained a small but significant factor (coefficient = –0.30, 95% CI: –0.59 to –0.01).

**Table 3.** Mixed-effects linear regression: Event-related and personal vulnerability covariates in relation to PTSS.

Variable	Coef.	Std error	*p*-value	95% CI	
Fixed effects					
Timepoint	−0.61	0.17	.00^a^	−0.95	−0.27
Days between timepoints^a^	0.00^a^	0.00^a^	.31	−0.00^a^	0.00^a^
Time since exposure to the most distressing PTE	−0.30	0.15	.04	−0.59	−0.01
Exposure to PTE					
Someone important to me was hospitalised due to COVID-19	Ref.^b^				
Own hospitalisation due to COVID-19	3.02	1.56	.05	−0.04	6.07
Someone important to me passed away from COVID-19	0.45	0.80	.56	−1.10	2.05
I was afraid that I or someone important to me would get COVID-19	−0.30	0.75	.77	−1.69	1.25
In my work, I saw many people who were seriously ill or passed away from COVID-19	−0.54	1.00	.71	−2.50	1.42
Due to COVID-19 measures, I was unable to provide support or care to someone important to me	2.17	0.82	.01	0.57	3.77
Due to COVID-19 measures, I was unable to say goodbye to someone who passed away	0.39	0.78	.62	−1.14	1.92
I experienced threats and/or violence due to discussions about COVID-19 measures	4.74	1.02	.00^a^	2.74	6.73
Age^c^					
Linear	0.08	0.25	.74	−0.40	0.56
Quadratic	0.00^a^	0.00^a^	.91	−0.01	0.01
Cubic^a^	−0.00^a^	0.00^a^	.57	−0.00^a^	0.00^a^
Loneliness^d^					
Not lonely	Ref.^b^				
Somewhat lonely	2.84	0.30	.00^a^	2.26	3.43
Very lonely	5.68	0.46	.00^a^	4.78	6.57
Stress^e^	2.48	0.16	.00^a^	2.17	2.80
Somatic symptoms^f^	1.54	0.09	.00^a^	1.36	1.73
Gender					
Men	Ref.^b^				
Women	−1.53	0.34	.00^a^	−2.10	−0.86
Education^g^					
Currently studying/going to school	Ref.^b^				
Low	0.83	1.02	.41	−1.17	2.82
Middle	0.72	0.98	.46	−1.18	2.64
High	0.10	0.92	.91	−1.69	1.89
Constant	7.22	3.42	.04	6.72	11.39
Random effects					
Between persons	58.34	3.17		52.45	64.89
Within-person	50.16	2.37		45.71	55.03

Note:

^a^ Value < 0.00 (not shown due to rounding).

^b^ Reference category.

^c^ Individual coefficients of the polynomial terms were not statistically significant, but the combined effect of these terms was significant (*p* < .001).

^d^ Loneliness measured with the short De Jong-Gierveld scale.

^e^ Count of factors causing the respondent to feel stressed (very) often (0–7).

^f^ Count of complaints (very) often experienced in the past 4 weeks (0–11).

^g^ Education levels are based on ISCED 2011. 0–2 = Low, 3–4 = Middle, 5–6 = High, ‘Currently in education’ = enrolled in any level of education (irrespective of ISCED level).

Personal vulnerability factors also played an important role. Loneliness was strongly associated with symptom severity: those who felt somewhat lonely reported higher PCL-5 scores (coefficient = 2.84, 95% CI: 2.26–3.43), and those who felt very lonely showed an even stronger association (coefficient = 5.68, 95% CI: 4.78–6.57), compared to participants who did not feel lonely. Perceived stress (coefficient = 2.48, 95% CI: 2.17–2.80) and somatic symptoms (coefficient = 1.54, 95% CI: 1.36–1.73) were positively related to symptom severity. Women reported lower symptom levels than men (coefficient = –1.53, 95% CI: –2.10 to –0.86). To explore nonlinear effects of age, age was modelled using linear, quadratic, and cubic terms. Although individual coefficients were not significant, a joint test showed that the age terms significantly improved model fit. Predicted PTSS severity followed a curvilinear pattern, peaking in middle adulthood (see Supplementary Figure 1).

The inclusion of these personal vulnerability factors substantially reduced the between-person variance to 58.34 (95% CI = 52.45–64.89), suggesting that a considerable portion of the interindividual differences in PTSD symptom severity can be explained by demographic and psychosocial characteristics. In contrast, the within-person variance remained virtually unchanged (50.16, 95% CI = 45.71–55.03), indicating that the added covariates did not account for intraindividual fluctuations in PTSD symptoms over time.

Model 2 demonstrated a substantially better fit than Model 1, as reflected by lower AIC (ΔAIC = −2704.73) and BIC (ΔBIC = −2631.80) values, and a higher log-likelihood (ΔLL = 1363.36), indicating improved explanatory power when accounting for personal vulnerability factors (Table 4).

**Table 4.** Model fit indices of model 1 and 2.

	Model 1	Model 2
AIC	44677.58	41972.85
BIC	44764.19	42132.39
LL	−22325.79	−20962.43

### 3.3. Mental wellbeing

The average MHI-5 score was 66.10 (SD = 17.84). 29.52% scored below the cut-off for mental problems (≤76). See the appendix for average prevalence of mental problems per round.

#### 3.3.1. Event related covariates

Multilevel linear regression models were used to assess the impact of event-related characteristics and personal vulnerability characteristics on mental wellbeing. In Model 3 (Table 5), which included only event-related covariates, a longer time since exposure to the most stressful COVID-19-related event was significantly associated with better mental wellbeing (B = 0.69, *p* < .001, 95% CI [0.27, 1.11]). Other variables, including the number of days between measurements and the measurement wave itself, were not significantly associated with mental wellbeing.

**Table 5.** Model 3: event-related covariates in relation to mental wellbeing.

Variable	Coef.	Std. error	*p*	95% CI	
Fixed effects					
Measurement	0.23	0.21	.27	−0.18	0.65
Days between measurements	0.00^a^	0.00^a^	.90	0.00^a^	0.00^a^
Time since exposure	0.69	0.21	.00^a^	0.27	1.11
Exposure					
Someone important to me was hospitalised due to COVID-19	Ref.^b^				
Own hospitalisation due to COVID-19	−2.36	2.80	.40	−7.84	3.13
Someone important to me passed away from COVID-19	−0.53	1.23	.66	−2.93	1.87
I was afraid that I or someone important to me would get COVID-19	−2.18	1.13	.05	−4.39	0.03
In my work, I saw many people who were seriously ill or passed away from COVID-19	−0.70	1.57	.66	−3.78	2.39
Due to COVID-19 measures, I was unable to provide support or care to someone important to me	−2.23	1.22	.07	−4.62	0.15
Due to COVID-19 measures, I was unable to say goodbye to someone who passed away	−0.77	1.20	.52	−3.13	1.58
I experienced threats and/or violence due to discussions about COVID-19 measures	−2.20	1.40	.12	−4.94	0.53
Constant	66.27	1.26	.00^a^	63.79	68.75
Random effects					
Between persons	221.57	7.61		207.15	237.00
Within-person	81.38	3.59		74.64	88.74

Note:

^a^ Value < 0.01 (not shown due to rounding).

^b^ Reference category.

Regarding the nature of exposure, participants who reported fear that they or someone important to them would contract COVID-19 reported marginally lower wellbeing compared to those whose close other had been hospitalised due to COVID-19 (B = −2.18, *p* = .05, 95% CI [−4.39, 0.03]). No other exposure types were significantly associated with wellbeing.

The between-person variance was estimated at 221.57 (95% CI = 207.15–237.00), while the within-person variance was 81.38 (95% CI = 74.64–88.74).

#### 3.3.2. Personal vulnerability factors

Model 4 (Table 6) included both event-related and personal vulnerability covariates in relation to mental wellbeing. Once personal vulnerability variables were added, time since exposure was no longer significantly associated with mental wellbeing (B = –0.04, *p* = .81), and none of the specific event-related exposures were significantly related to wellbeing.

**Table 6.** Model 4: event-related and personal vulnerability covariates in relation to mental wellbeing.

Variable	Coef.	Std error	*p*-value	95% CI	
Fixed effects					
Timepoint	−0.16	0.19	.38	−0.53	0.20
Days between timepoints	0.00^a^	0.00^a^	.79	−0.00^a^	0.00^a^
Time since exposure to the most distressing PTE	−0.04	0.16	.81	−0.34	0.27
Exposure to PTE					
Someone important to me was hospitalised due to COVID-19	Ref.^b^				
Own hospitalisation due to COVID-19	−1.44	2.12	.50	−5.59	2.71
Someone important to me passed away from COVID-19	0.39	0.87	.65	−1.32	2.10
I was afraid that I or someone important to me would get COVID-19	−0.44	0.79	.57	−2.00	1.11
In my work, I saw many people who were seriously ill or passed away from COVID-19	1.23	1.07	.25	−0.87	3.34
Due to COVID-19 measures, I was unable to provide support or care to someone important to me	0.76	0.87	.38	−0.94	2.46
Due to COVID-19 measures, I was unable to say goodbye to someone who passed away	1.07	0.85	.21	−0.59	2.74
I experienced threats and/or violence due to discussions about COVID-19 measures	1.14	1.03	.27	−0.87	3.16
Age^c^					
Linear	−0.29	0.25	.25	−0.78	0.20
Quadratic	0.01	0.00^a^	.08	−0.00^a^	0.02
Cubic	−0.00^a^	0.00^a^	.05	−0.00^a^	0.00^a^
Loneliness^d^					
Not lonely	Ref.^b^				
Somewhat lonely	−5.23	0.36	.00^a^	−5.93	−4.52
Very lonely	10.75	0.51	.00^a^	11.76	−9.74
Stress^e^	−3.88	0.16	.00^a^	−4.20	−3.56
Somatic symptoms^f^	−2.13	0.10	.00^a^	−2.32	−1.93
Gender					
Men	Ref.^b^				
Women	−1.05	0.38	.01	−1.80	−0.31
Education^g^					
Currently studying/going to school	Ref.^b^				
Low	−1.77	1.06	.10	−3.86	0.32
Middle	−1.09	1.00	.28	−3.05	0.87
High	−0.84	0.97	.39	−2.73	1.06
Constant	82.59	3.42	.00^a^	75.89	89.30
Random effects					
Between persons	67.24	3.44		60.72	74.24
Within-person	62.97	2.90		57.54	68.91

Note:

^a^ Value < 0.00 (not shown due to rounding).

^b^ Reference category.

^c^ Individual coefficients of the polynomial terms were not statistically significant, but the combined effect of these terms was significant (*p* < .001).

^d^ Loneliness measured with the short De Jong-Gierveld scale.

^e^ Count of factors causing the respondent to feel stressed (very) often (0–7)

^f^ Count of complaints (very) often experienced in the past 4 weeks (0–11)

^g^ Education levels are based on ISCED 2011. 0–2 = Low, 3–4 = Middle, 5–6 = High, ‘Currently in education’ = enrolled in any level of education (irrespective of ISCED level).

In contrast, several personal vulnerability factors showed strong and consistent associations. A clear gradient emerged for loneliness: participants who reported being somewhat lonely had significantly lower wellbeing (B = –5.23, *p* < .001), and those who felt very lonely reported even lower scores (B = –10.75, *p* < .001), compared to individuals who did not feel lonely. Higher perceived stress (B = –3.88, *p* < .001) and more somatic symptoms (B = –2.13, *p* < .001) were also linked to poorer wellbeing. Women reported slightly lower wellbeing than men (B = –1.05, *p* = .01). To assess potential nonlinear effects of age, linear, quadratic, and cubic terms were included. A joint significance test indicated that age had a significant curvilinear association with mental wellbeing (*p* < .001), despite the individual terms being only marginally significant. Predicted values showed that mental wellbeing was lowest at younger ages, increased into mid-to-late adulthood, and declined again in older age, suggesting the highest levels of wellbeing occurred in midlife (see Supplementary Figure 2).

The inclusion of these additional factors substantially reduced the between-person variance to 67.24 (95% CI = 60.72–74.24), indicating that demographic and psychosocial characteristics explained a large proportion of the interindividual variation in mental well-being. By contrast, the within-person variance remained similar (62.97, 95% CI = 57.54–68.91), suggesting that the additional factors captured stable differences between individuals but did not account for fluctuations across time within individuals.

Model 4, which included personal vulnerability factors, demonstrated a substantially better fit than Model 3, as indicated by lower AIC (ΔAIC = −4369.99) and BIC (ΔBIC = −4297.07) values, and a higher log-likelihood (ΔLL = 2195.99), reflecting improved explanatory value (Table 7).

**Table 7.** Model fit indices of model 3 and 4 for mental wellbeing.

	Model 3	Model 4
AIC	47457.21	43087.22
BIC	47543.82	43246.75
LL	−23715.60	−21519.61

### 3.4. Sensitivity analysis

Across all three time-varying vulnerability factors (loneliness, stress, and somatic complaints), cross-lagged analyses indicated bidirectional associations with both PTSS and mental well-being. However, the strength of these associations was not symmetrical: effects from vulnerability factors to subsequent mental health outcomes were generally larger than the reverse effects from prior mental health status to later loneliness, stress and somatic complaints. These results are presented in Supplementary Table 1.

### 3.5. Impact of the COVID-19 pandemic on processing events

In total, 488 participants provided a response to the open-ended question. The majority of these responses were excluded from analysis because they were either too vague or not directly relevant to the question. Consequently, 193 responses were deemed suitable for coding and included in the analysis. Participants often described the COVID-19 pandemic as complicating the processing of potentially traumatic events, though some also noted positive effects. Four main themes emerged: lack of social contact and support, disruption of mourning rituals, fear and uncertainty, and renewed appreciation for life and relationships.

Many participants reported feelings of loneliness and isolation. Everyday activities that normally provided social connection, such as attending church or caring for others, were often unavailable, making it harder to share experiences or receive support.

Not being able to talk to someone about it in person or seek closeness.

Negative. We were in lockdown, and I wasn’t able to have contact with the people I needed at that time.

Experiences of loss were often described as particularly difficult. Several participants mentioned the inability to properly say goodbye to loved ones, which impeded their grieving process. Traditional mourning rituals, such as funerals or gatherings with friends and family, were often disrupted or not possible. This lack of closure contributed to feelings of helplessness and prolonged sorrow.

My grandmother was dying but we could not say goodbye. On top of that, it was hard not being able to hug family during the funeral.

The pandemic was marked by persistent uncertainty and fear, of infection, of the unknown trajectory of the pandemic, and of its long-term consequences. These ongoing fears created a background of tension and caution that many participants felt obstructed their emotional processing.

I am constantly afraid that this person catches COVID and might not survive.

Because of the ongoing uncertainty surrounding COVID-19, it is difficult to move past the fear.

Despite the challenges, several participants reflected on the pandemic as a moment of existential awareness. The disruption of normal life prompted re-evaluation of personal values, life goals, and priorities. In some cases, this led to a renewed appreciation for relationships with loved ones, their own health and the health of their loved one, or how short life is, improving the processing of the event.

Due to the COVID period and the lockdowns, I started reflecting more, reached out to my grandparents abroad more often, and our bond has grown stronger. It has had a positive impact.

## 4. Discussion

The results from this study show that trauma-related mental health after COVID-19-related PTEs was initially correlated with both event-related and personal vulnerability risk factors. For PTSS, symptoms decrease over time, but this effect is smaller when personal vulnerability factors are included in the model. Similarly, type of event shows the same pattern: some events (hospitalisation due to COVID-19, being unable to provide care or support to a loved one, and experiencing violence or threats related to discussions about COVID-19 measures) were associated with higher PTSS, but these effects were reduced when personal vulnerability factors were added. For mental wellbeing, longer time since exposure was significant in the simpler model, but this effect disappeared in the full model including personal vulnerability factors. These findings suggest that while event-related characteristics initially appear relevant, individual vulnerability factors are the primary covariates in relation to both PTSS and general mental wellbeing when considered simultaneously. Especially for mental wellbeing, event-related factors play no role in the more extensive model.

It is plausible that event-related characteristics play a more prominent role in PTSS than in overall mental wellbeing because PTSS is inherently tied to specific traumatic events. Posttraumatic stress disorder is the only mental disorder in the DSM-5 for which exposure to a traumatic event is a diagnostic criterion (APA, 2013). PTSS reflect how individuals interpret and cope with that specific event. In contrast, mental wellbeing represents a broader evaluation of psychological functioning, shaped by a wider array of factors, including personality, social context, and long-term stressors. Consequently, characteristics of the traumatic event account for more variation in PTSS than in general mental wellbeing, whereas individual factors largely drive overall mental health outcomes.

The findings indicate bidirectional associations between vulnerability factors (loneliness, perceived stress, and somatic complaints) and both PTSS and mental well-being, suggesting dynamic feedback processes over time. However, these associations were asymmetric, with stronger effects from vulnerability factors to subsequent mental health than vice versa. This may indicate that vulnerability factors are more important drivers of changes in mental health, whereas reverse effects are weaker.

Our findings are consistent with research suggesting that the disaster context in which a traumatic event occurs plays an important role in the development of mental health problems (Bonanno et al., 2010; Tortella-Feliu et al., 2019). The interplay between the individual, the traumatic event, and the surrounding context reflects a complex dynamic consistent with the ecological model (Bronfenbrenner, 1979; Harvey, 1996), which emphasises that trauma-related mental health outcomes are shaped not only by characteristics of the person or the event itself, but also by the broader environment in which the trauma occurs.

In our models, we found that the inclusion of demographic and psychosocial factors substantially reduced the variance between individuals in both PTSD symptoms and mental well-being, whereas the within-person variance remained largely unchanged. This pattern suggests that these variables, such as loneliness, stress, and somatic symptoms, account for considerable interindividual differences in mental health outcomes, but that theydo not explain why an individual’s symptoms fluctuate over time. This finding resonates with Bonanno’s resilience paradox (Bonanno, 2021), which highlights that resilience is common, yet difficult to predict at the individual level. Although certain risk and protective factors consistently distinguish groups with higher or lower average symptom levels, they do not fully capture the dynamic, situational, and often complex nature of individual trajectories. The results similarly show that while demographic and psychosocial characteristics clarify who is more vulnerable or resilient, they fail to illuminate when and why an individual’s mental health changes over time.

The COVID-19 pandemic created a uniquely challenging context for processing traumatic events. The qualitative findings from our study revealed several themes in which coping is hindered by the COVID-19 pandemic, which are consistent with other literature and our quantitative findings. In particular, many participants described how lockdowns and social distancing restricted access to social support and led to isolation. Especially social support is associated with recovery from psychological distress after a traumatic event (Birkeland et al., 2017; Sippel et al., 2015, 2024). This is consistent with the strong association between loneliness and mental health outcomes in the quantitative models.

In the context of the loss of loved ones during previous pandemics, some studies have found that restricted or absent funerals and grief rituals can have a negative impact on the grieving process and adaptation (Mayland et al., 2020). Previous studies suggest that COVID-19-related stressors such as being unable to attend a funeral, say goodbye, or experiencing changes in funeral services may be associated with higher levels of psychopathology (Reitsma et al., 2023). Experiences of disrupted mourning and the inability to say goodbye to loved ones further illustrate how pandemic-related restrictions may have prolonged emotional processing and hindered recovery. This may help explain why time since exposure showed limited effects in the full models, as conditions that typically facilitate adaptation and closure were often absent during the pandemic period.

Ongoing uncertainty and fear of infection can further hinder recovery according to the participants. This aligns with other studies showing that fear of infection is associated with mental health problems, mainly anxiety and depression, but also with PTSS in some cases (Alimoradi et al., 2022; Rabiu Abubakar et al., 2022). This aligns with the quantitative finding that general stress was a strong covariate for both PTSS and well-being. The pandemic context may have led to prolonged stress experiences, that influence coping and recovery.

While some found space for reflection and connection during the pandemic, most participants described the pandemic as a period that significantly complicated emotional processing, setting it apart from other post-trauma contexts. These stressors may help explain why trauma-related symptoms did not decrease much over time in our study, as the conditions that typically support recovery were impaired throughout the pandemic.

### 4.1. Strengths and limitations

This study has several strengths. First, we conducted a detailed assessment of event-related characteristics. Unlike many large-scale population studies conducted during the COVID-19 pandemic that rely on general trauma screening (van Duinkerken et al., 2025), this study explicitly asked participants to indicate which type of potentially traumatic event they experienced during the COVID-19 pandemic and how long ago the event took place. This allowed for a more precise estimation of the effects of specific types of exposure and enabled the inclusion of time since exposure as a covariate in the models.

Second, the longitudinal design allowed for the examination of changes in symptoms within individuals over time. This is particularly valuable in the context of trauma-related outcomes, which may evolve as individuals process events. Repeated assessments further enabled adjustment for the time between the event and the measurement and allowed for a more nuanced understanding of symptom development compared to cross-sectional studies.

Third, the study was conducted within a large, population-based cohort during a global public health crisis. This provides a unique opportunity to follow the impact of trauma exposure during such a crisis over time and to identify psychosocial and demographic factors associated with vulnerability or resilience under real-world conditions.

Despite these strengths, several limitations should be considered when interpreting the findings.

The study focused on a predefined set of pandemic-related traumatic events, which may not have captured all relevant stressors experienced by participants, potentially limiting the comprehensiveness of the assessment of event-related influences on mental health outcomes.

The inclusion of personal vulnerability risk factors was constrained by the structure of the survey. Not all psychosocial or demographic variables were assessed consistently across all measurement waves, and some measures were only included in either the adult or adolescent version of the survey. This restricted the number of covariates that could be included in the adjusted models.

Individuals who were exposed to potentially traumatic events but did not endorse ongoing distress in response to the screening question (‘are you still affected by this event?’) were not administered the PCL-5. As a result, individuals who may have experienced transient PTSS or who had already recovered at the time of the assessment were excluded. This may have led to an overestimation of symptom severity among those exposed and limited the ability to examine early recovery processes.

The analyses of mental well-being (MHI-5) were restricted to participants who also completed the PCL-5 and reported still being affected by a pandemic-related event. This decision was made to ensure consistency in the analytic sample and to examine mental well-being within the context of ongoing event-related distress, as the PCL-5 was only administered to this subgroup. However, this restriction may have introduced selection bias, as inclusion in this subsample is likely influenced by both event severity and individual vulnerability. As a result, associations between event-related factors and well-being may have been attenuated, and the relative importance of personal vulnerability factors may have been overestimated. This should be taken into account when interpreting conclusions regarding the dominant role of personal vulnerability in explaining mental well-being.

For participants who were still enrolled in education, we did not distinguish between educational levels. Since their final level of education was unknown, categorising them prematurely could have been misleading. However, this approach resulted in a heterogeneous group, which may have diluted potential associations and contributed to some non-significant results.

Although the study included multiple measurement points, there was considerable variation in the time between exposure and the first assessment across participants. In many cases, the first measurement occurred several months after the most distressing event, meaning that the baseline level of symptoms may already have been influenced by natural recovery or other intervening factors. While we statistically adjusted for both the time since the event and the time between measurement waves, such heterogeneity may have affected the comparability of symptoms over time across individuals.

Within-person trajectories were not examined, as the data structure did not allow for robust modelling of within-person change over time. Consequently, the analyses primarily reflect between-person differences, which limits the ability to draw conclusions about individual-level symptom dynamics.

Perceived stress was assessed using a study-specific set of items rather than a fully validated standardised scale. Although the measure captures multiple sources of stress and provides a broad indication of perceived stress levels, the lack of a validated instrument may limit the comparability and psychometric robustness of this construct and should be taken into account when interpreting the findings.

Open-ended responses were only collected in a single measurement round. As a result, these data could not be incorporated into the longitudinal models, which required repeated measures across time. While the qualitative input provides valuable contextual insight, the lack of repeated assessment limits the ability to examine changes in coping mechanisms over time. Additionally, many responses were excluded due to being vague or irrelevant, which may have resulted in attrition in the qualitative responses and potential selection bias in the analysed subsample, as the retained responses may not fully represent the range of experiences among all participants.

A final limitation of this study is that the data were collected in the Netherlands, which may limit generalizability to other cultural and geographical contexts, given differences in pandemic responses, healthcare systems, and social policies. However, prior international evidence using WHO Mental Health Survey data (Dückers & Brewin, 2016; Ruscio et al., 2017; Stein et al., 2017) and Global Burden of Disease data (Choi et al., 2024; Javaid et al., 2023) suggests substantial mental health burden across settings, with higher estimates often observed in higher-income or more developed countries, including during the COVID-19 pandemic (Bosmans et al., 2025; Fan et al., 2025). Future research should replicate these findings in other contexts and crisis types.

### 4.2. Implications

This study shows that while both event-related and personal vulnerability risk factors are associated with trauma-related mental health after COVID-19-related events, personal vulnerability factors have a stronger impact. Responses to the open-ended questions further illustrate these points, highlighting how participants felt like missing social connection and support hindered their coping after exposure to PTEs during the pandemic. The findings support ecological models of trauma, which emphasise the dynamic interplay between event characteristics and individual vulnerabilities (Harvey, 1996). This underscores the need for interventions that go beyond the traumatic event itself and focus on broader psychosocial support, such as strengthening social networks and reducing overall stress, which may enhance resilience and be more effective for long-term recovery and mental wellbeing than event-focused approaches.

This study provides insight into vulnerabilities for higher PTSS and lower mental wellbeing, such as loneliness and stress, and these vulnerabilities were confirmed by the open-ended responses, which highlighted the role of lacking social support and feelings of fear and uncertainty. These insights are particularly relevant for future pandemic preparedness: health responses should not only address the acute impact of potentially traumatic events but also proactively invest in structural and social supports that can buffer long-term psychological harm, especially in vulnerable groups. During future pandemics or crises, it is crucial to avoid prolonged social disconnection and to safeguard access to emotional and social support, even under restrictive measures, to better support psychological recovery.

Future research should further examine the relative contributions of event-related and individual vulnerability factors to trauma-related mental health, using longitudinal designs with earlier assessments to capture symptom trajectories and natural recovery processes. Including individuals who experience transient PTSS would allow for a better understanding of early recovery. Additionally, future research should build on the explorative qualitative findings by systematically examining how large-scale public health crises such as the COVID-19 pandemic disrupt normal coping processes and recovery.

Although this study is situated within the context of the COVID-19 pandemic, the identified associations may extend to other large-scale crises, such as natural disasters and future public health emergencies. This broader relevance underscores the continued importance of COVID-19-related mental health research, as it provides insight into general mechanisms of psychological vulnerability and resilience under conditions of widespread societal disruption. Future crisis responses should therefore not only focus on the immediate impact of traumatic events, but also proactively protect and strengthen social and emotional support systems and proactively improve wellbeing in order to mitigate long-term psychological harm.

## Supplementary Material

Supplemental Material

## Data Availability

The data used in this study are not publicly available but can be requested (in Dutch) through the website of the consortium, upon reasonable request and subject to approval. https://www.rivm.nl/gor-covid-19/onderzoeksdata-aanvragen.
