# Supplementary material for: Exploring the impact of event-related and personal vulnerability characteristics on mental health after pandemic-related trauma exposure
Source: Eur J Psychotraumatol. 2026 Jul 31;17(1):2700829. doi: 10.1080/20008066.2026.2700829 (PMC13431024; doi:10.1080/20008066.2026.2700829)
Supplement: Supplemental Material [file ZEPT_A_2700829_SM5782.docx]

Supplementary Table 1

| **Outcome (t+1)** | **Predictor (t)** | **B** | **95% CI** | **p** |
| --- | --- | --- | --- | --- |
| **Trauma and Loneliness** |  |  |  |  |
| Loneliness | Loneliness | 0.626 | 0.407 to 0.844 | < .001 |
| Loneliness | PTSS | 0.016 | 0.012 to 0.021 | < .001 |
| PTSS | PTSS | 0.679 | 0.633 to 0.725 | < .001 |
| PTSS | Loneliness | 0.734 | 0.702 to 0.766 | < .001 |
| **Trauma and somatic complaints** |  |  |  |  |
| Somatic complaints | Somatic complaints | 0.708 | 0.667 to 0.749 | < .001 |
| Somatic complaints | PTSS | 0.015 | 0.008 to 0.021 | < .001 |
| PTSS | PTSS | 0.679 | 0.634 to 0.724 | < .001 |
| PTSS | Somatic complaints | 0.324 | 0.106 to 0.541 | .004 |
| **MHI-5 and Loneliness** |  |  |  |  |
| MHI-5 | MHI-5 | 0.716 | 0.706 to 0.726 | < .001 |
| MHI-5 | Loneliness | –0.832 | –0.967 to –0.696 | < .001 |
| Loneliness | Loneliness | 0.715 | 0.706 to 0.725 | < .001 |
| Loneliness | MHI-5 | –0.012 | –0.014 to –0.010 | < .001 |
| **Model 4: MHI-5 and SOLK** |  |  |  |  |
| MHI-5 | MHI-5 | 0.707 | 0.697 to 0.717 | < .001 |
| MHI-5 | Somatic complaints | –0.878 | –1.029 to –0.728 | < .001 |
| Somatic complaints | Somatic complaints | 0.637 | 0.623 to 0.650 | < .001 |
| Somatic complaints | MHI-5 | –0.015 | –0.016 to –0.013 | < .001 |
| **Model 5: MHI-5 and Stress** |  |  |  |  |
| MHI-5 | MHI-5 | 0.698 | 0.687 to 0.709 | < .001 |
| MHI-5 | Stress | –1.613 | –1.894 to –1.329 | < .001 |
| Stress | Stress | 0.518 | 0.501 to 0.536 | < .001 |
| Stress | MHI-5 | –0.015 | –0.016 to –0.013 | < .001 |
| **Model 6: Trauma and Stress** |  |  |  |  |
| Stress | Stress | 0.582 | 0.528 to 0.635 | < .001 |
| Stress | PTSS | 0.015 | 0.010 to 0.020 | < .001 |
| PTSS | PTSS | 0.679 | 0.634 to 0.724 | < .001 |
| PTSS | Stress | 0.474 | 0.118 to 0.831 | .009 |

Supplementary Figure 1


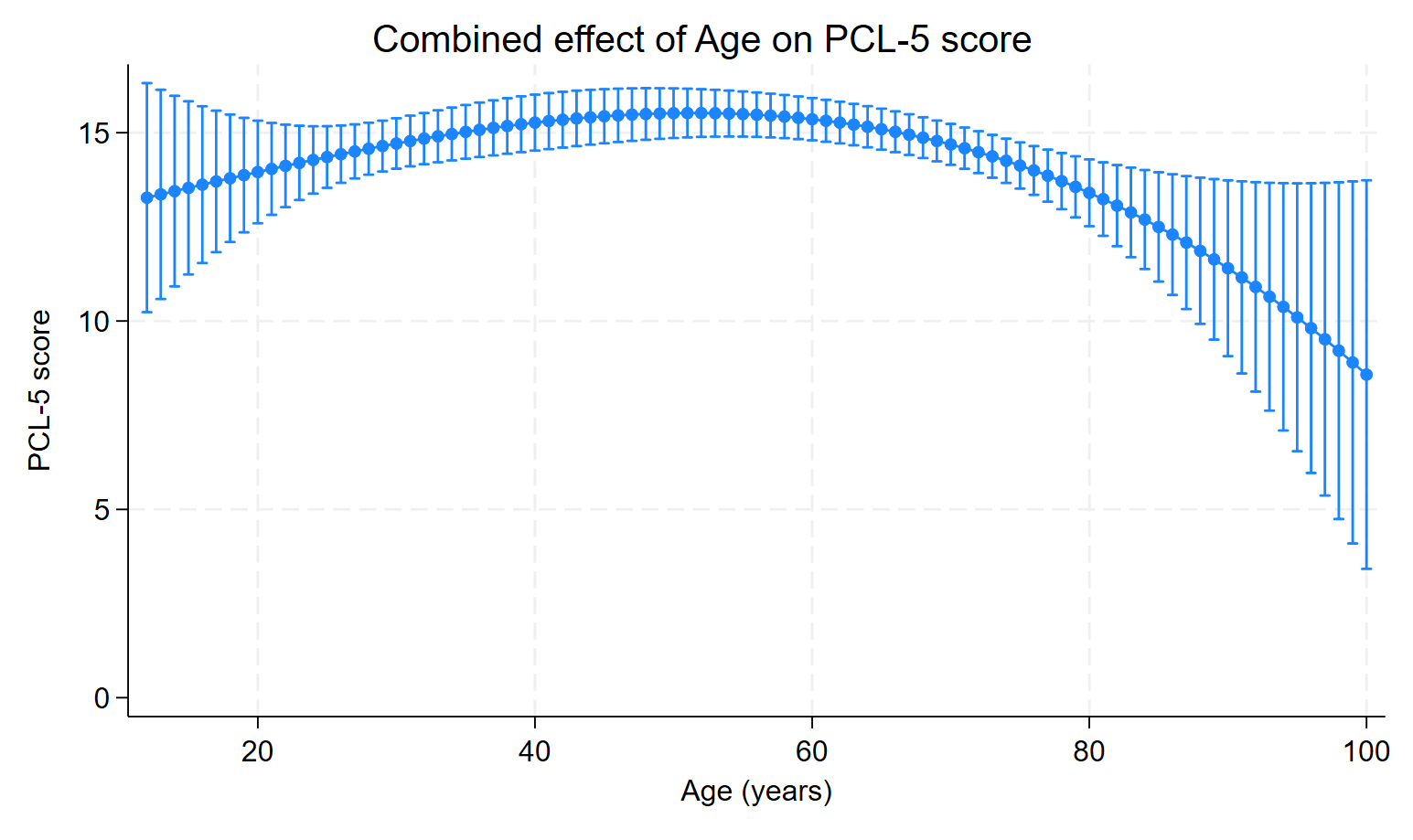


Supplementary Figure 2
